# TMT-based quantitative proteomics analysis reveals the key proteins related with the differentiation process of goat intramuscular adipocytes

**DOI:** 10.1186/s12864-021-07730-y

**Published:** 2021-06-05

**Authors:** Yu Du, Yong Wang, Qing Xu, Jiangjiang Zhu, Yaqiu Lin

**Affiliations:** 1 Key Laboratory of Qinghai-Tibetan Plateau Animal Genetic Resource Reservation and Utilization of Education Ministry, Southwest Minzu University, Chengdu, China; 2 Key Laboratory of Qinghai-Tibetan Plateau Animal Genetic Resource Reservation and Exploitation of Sichuan Province, Southwest Minzu University, Chengdu, China; 3 College of Animal & Veterinary Science, Southwest Minzu University, Chengdu, China

**Keywords:** Proteomic, Differentially abundant proteins, Intramuscular preadipocytes, Differentiation, Tandem mass tag, Parallel Reaction Monitoring

## Abstract

**Background:**

Intramuscular adipocytes differentiation is a complex process, which is regulated by various transcription factor, protein factor regulators and signal transduction pathways. However, the proteins and signal pathways that regulates goat intramuscular adipocytes differentiation remains unclear.

**Result:**

In this study, based on nanoscale liquid chromatography mass spectrometry analysis (LC-MS/MS), the tandem mass tag (TMT) labeling analysis was used to investigate the differentially abundant proteins (DAPs) related with the differentiation process of goat intramuscular adipocytes. Gene Ontology, Kyoto Encyclopedia of Genes and Genomes enrichment and protein-protein interaction network analyses were performed for the characterization of the identified DAPs. The candidate proteins were verified by parallel reaction monitoring analysis. As a result, a total of 123 proteins, 70 upregulation proteins and 53 downregulation proteins, were identified as DAPs which may be related with the differentiation process of goat intramuscular adipocytes. Furthermore, the cholesterol metabolism pathway, glucagon signaling pathway and glycolysis / gluconeogenesis pathway were noticed that may be the important signal pathways for goat Intramuscular adipocytes differentiation.

**Conclusions:**

By proteomic comparison between goat intramuscular preadipocytes (P_IMA) and intramuscular adipocytes (IMA), we identified a series protein that might play important role in the goat intramuscular fat differentiation, such as SRSF10, CSRP3, APOH, PPP3R1, CRTC2, FOS, SERPINE1 and AIF1L, could serve as candidates for further elucidate the molecular mechanism of IMF differentiation in goats.

**Supplementary Information:**

The online version contains supplementary material available at 10.1186/s12864-021-07730-y.

## Background

Intramuscular fat (IMF) is the adipose tissue between muscle fibers, also known as marbling. IMF content has a positive effect on meat tenderness, moisture content and palatability [1–4]. As an important economic trait of lamb production, reasonable IMF content can create greater economic benefits and further improve the taste and quality of meat [2, 5].

Adipose tissue is mainly composed of a large number of adipocytes, which are generated through the proliferation and differentiation of preadipocytes. Study showed that the preadipocytes are present throughout adult life, exhibiting adipose depot specificity [6]. In addition, the differentiation of preadipocytes into adipocytes is a complex process, which is regulated by various transcription factor, protein factor regulators and signal transduction pathway [6–8]. In porcine preadipocytes, miR-429 inhibits subcutaneous and intramuscular preadipocytes differentiation while promotes proliferation by directly binding to the 3′-UTRs of *KLF*9 and *p*27 [9]. Emerging research on preadipocyte differentiation using proteome and transcriptome analysis better revealed the breadth of adipocyte differentiation. For instance, using HPLC-tandem mass spectrometry and methylated RNA immunoprecipitation (meRIP), found that MTCH2 promotes adipogenesis in pig P_IMA via an m6A-YTHDF1-dependent mechanism [10].

The differential expression of cellular proteins affects the cell state. Studies have showed that the relationship between protein and mRNA expression levels is a comprehensive result of translation and protein degradation, while the genome-wide correlation between mRNA expression levels and proteins is very low, and study showed that only about 40 % of protein expression differences can use the change of transcription to explain [11–13]. However, the significant difference in mRNA expression between conditions is often used for biological discovery. Moreover, many measurements on mRNA can be traced back to comparison with protein data, that is, attributing functional differences between conditions to protein action [14–16]. Adipocyte differentiation are complex quantitative traits that is regulated by multiple genes and proteins. Transcriptome and proteomics analysis of intramuscular adipocyte differentiation and fat deposition are critical to goat meat production and its quality. However, the key regulatory proteins in differentiation of goat intramuscular adipocytes still unclear.

Here, we used Tandem Mass Tags (TMT) quantitative proteomics method to analyze and screen the different-expressed proteins between goat P_IMA and IMA. Also, Parallel Reaction Monitoring (PRM) technology was used for validating the quantitative analysis of differentially expressed proteins. The results are of great significance for elucidating the molecular mechanism of IMF differentiation in goats.

## Results

### Identification of goat P_IMA and IMA differentiation model

**Fig. 1:**
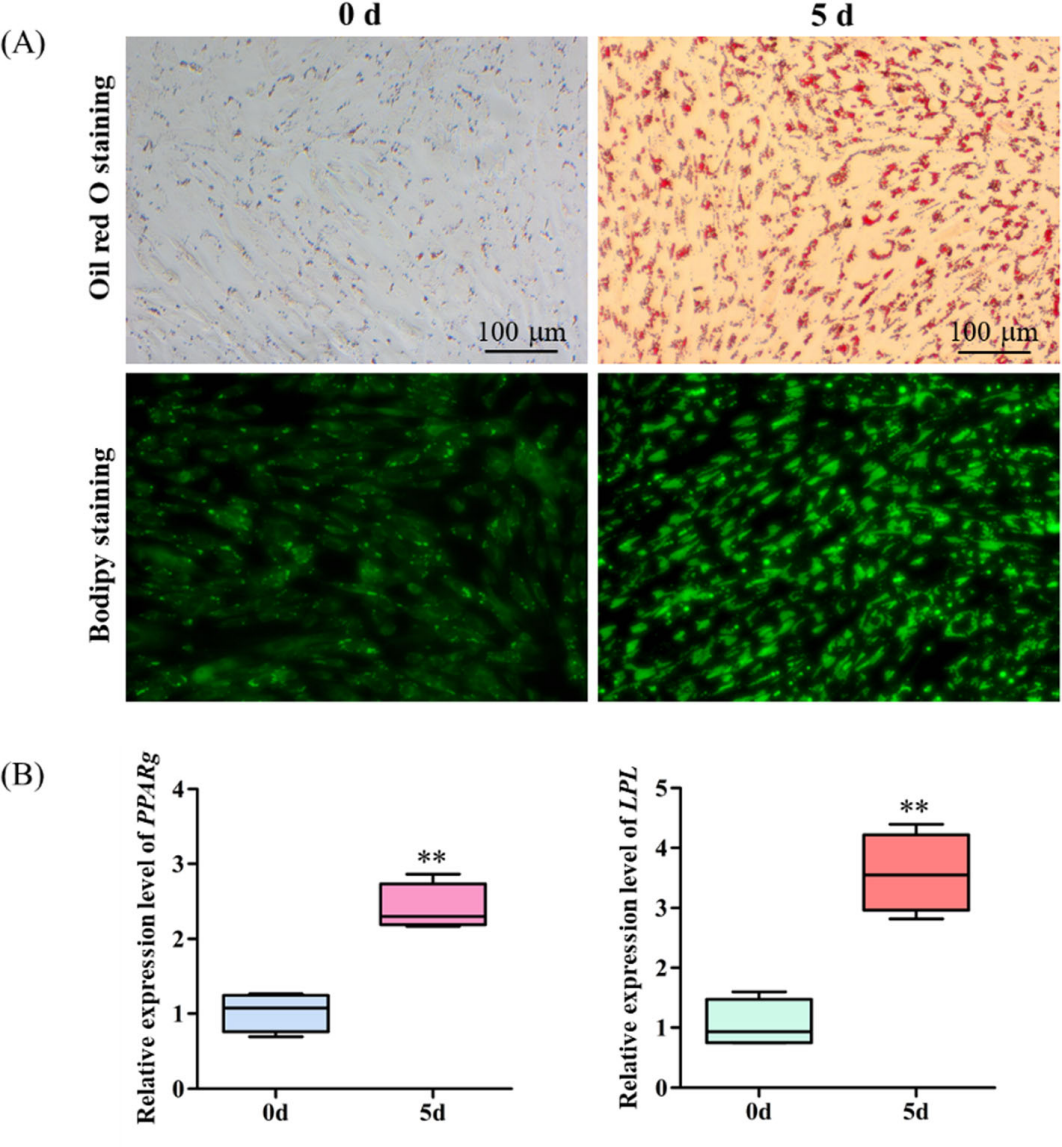
Differentiation of intramuscular preadipocytes induced in vitro. **A** Oil Red O staining and Bodipy staining of P_IMA and IMA. **B** The mRNA expression levels of key adipogenic regulatory genes detected by qRT-PCR. Data are shown as mean ± SD of four independent experiment. *P < 0.05; **P < 0.01

Goat IMA differentiation model in vitro was constructed in this study. As shown in Fig. 1A, cells were filled with lipid droplets after 5 days induction. Furthermore, the results of quantitative real time PCR (qRT-PCR) showed that expression of key adipogenic differentiation genes *PPARg* and *LPL* were significantly up-regulated (Fig. 1B).

### Protein identification and quantitative proteome analysis based on TMT

**Fig. 2:**
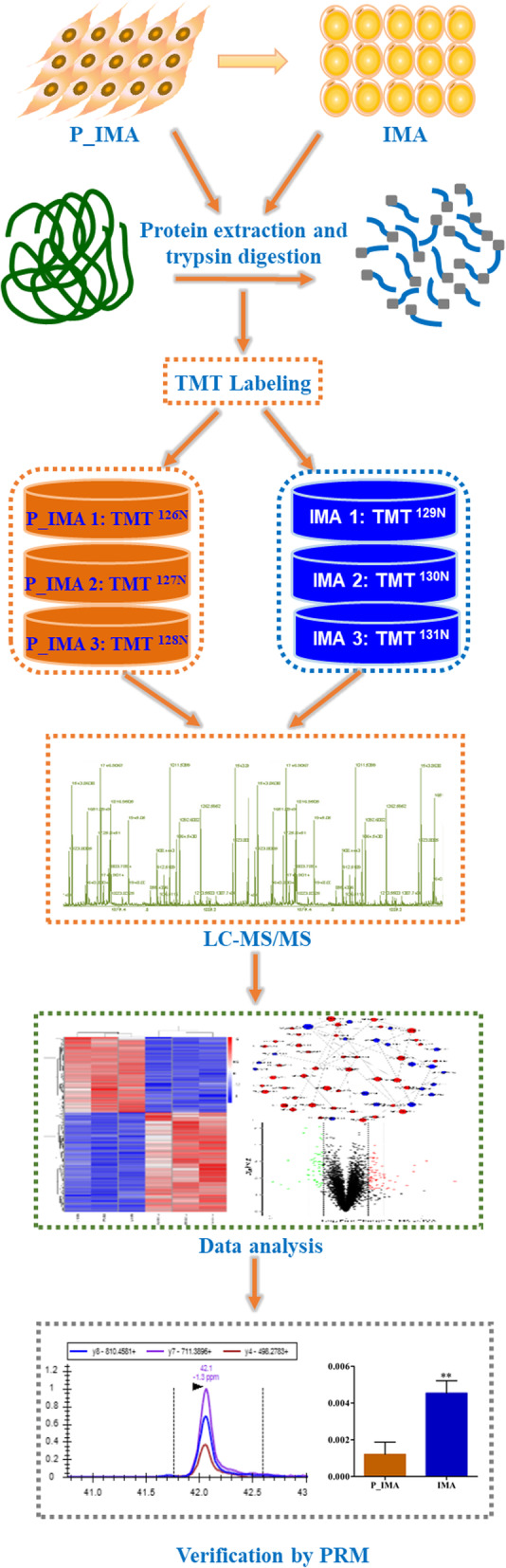
Schematic diagram of the experimental flow. The P_IMA indicates the intramuscular preadipocytes and the IMA indicates the intramuscular adipocytes. The solid arrow shows the differentiation process from intramuscular preadipocytes to adipocytes, while the dotted arrows show the experimental flow. Diagram detailing the experiment including the principle, sample model establishment, data acquisition and data analysis

Three samples of goat P_IMA, induced 0d, and IMA, induced 5d, respectively, were analyzed using TMT proteomics technique to identify the differentially abundant proteins (DAPs). And the experimental strategy is shown in Fig. 2.

**Fig. 3:**
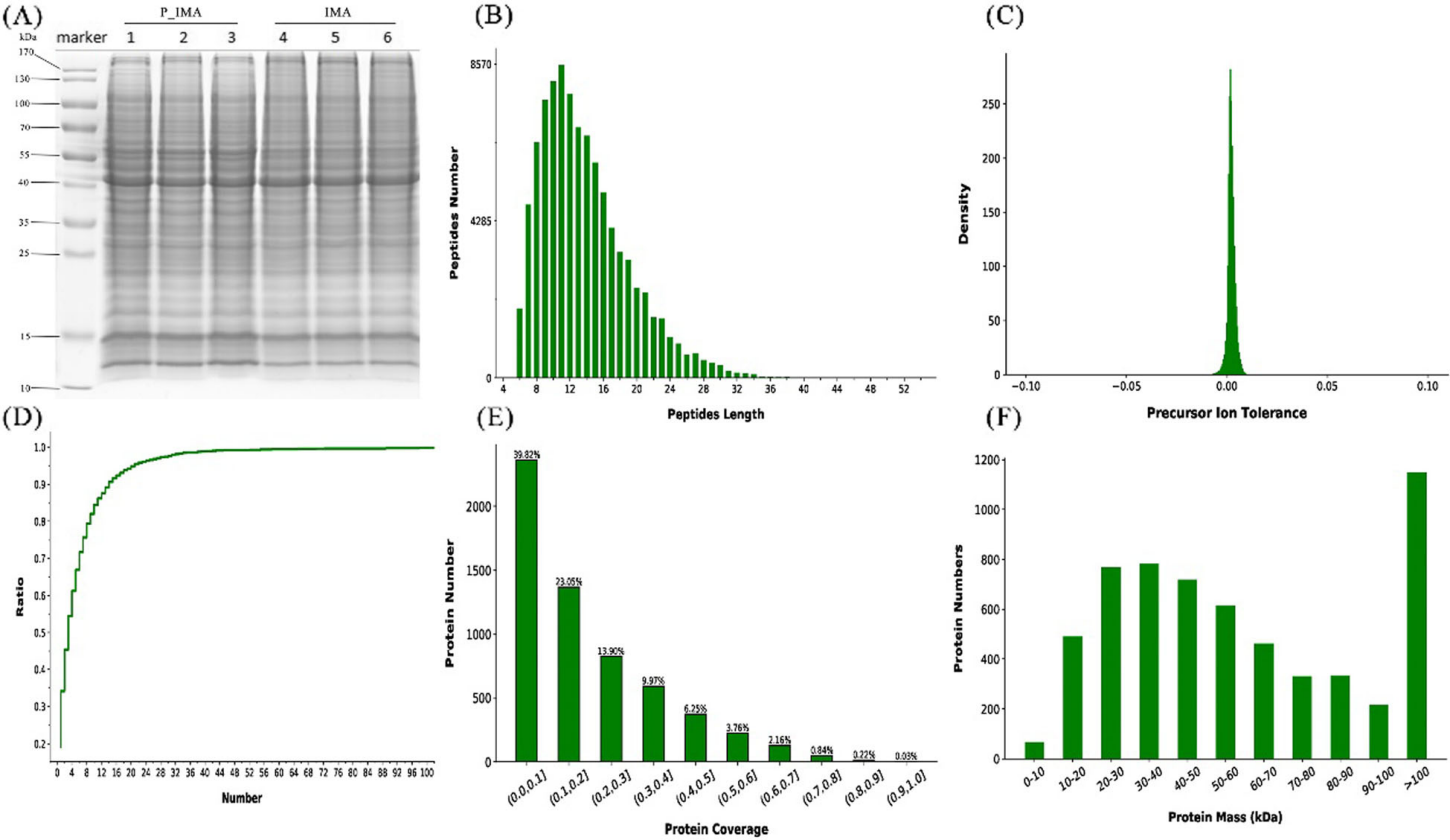
Quality control results of the proteins. **A** The SDS-PAGE electrophoresis diagram. **B** Verification of the total protein quality, the abscissa represents the number of amino acid residues in the peptide, and the ordinate represents the number of peptides of this length. **C** Precursor ion tolerance, the abscissa represents the mass deviation, and the ordinate represents the precursor ion density distribution of the corresponding error. **D** Unique peptides number, slow increase of the curve means more unique peptides. **E** protein coverage, the abscissa represents the protein coverage interval, and the ordinate represents the number of proteins contained in the interval (**F**) Protein mass distribution, the abscissa represents the molecular weight of the identified protein, and the ordinate represents the number of the identified protein. A large molecular weight range indicates a wide range of identified proteins

**Fig. 4:**
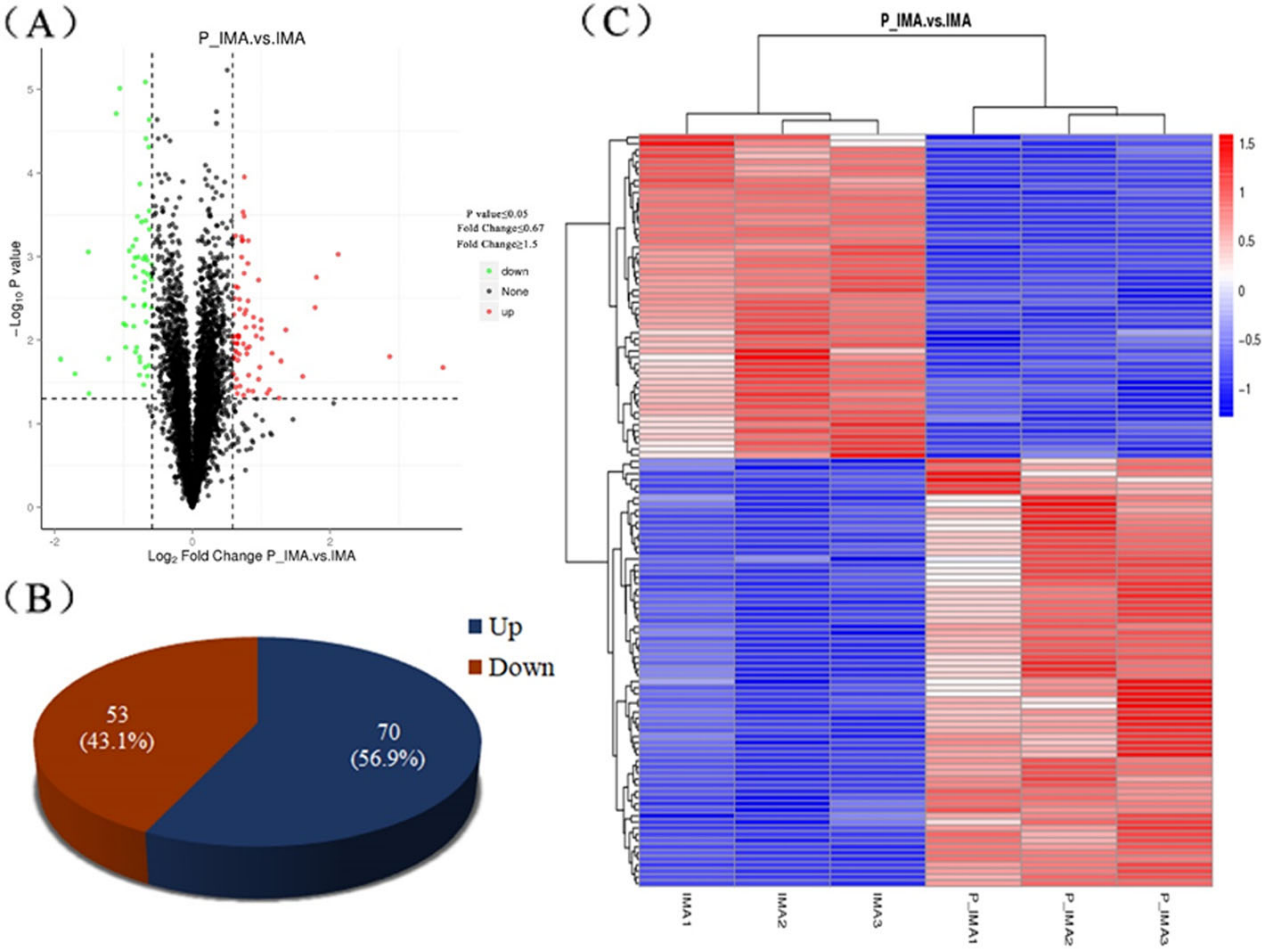
The analysis of identified proteins. **A** The volcano plot of identified proteins between P_IMA and IMA. **B** The distribution of up-regulated and down-regulated proteins in the DAPs that we obtained. **C** Hierarchical cluster analysis of relative protein content of the protein samples

Protein we selected containing at least one unique peptide, and the false discovery rate (FDR) of the peptide and protein were < 1 %. In this study, a total of 5929 proteins were quantified that were jointly found in two samples (Supplementary material S1). The quality control results of the proteins were showed in Fig. 3. For comparison between the intramuscular adiposes samples between 0d and 5 day, the proteins’ difference multiple (log2 value) and p-value (-log10 value) were used as the abscissa and ordinate to generate the difference protein volcano map (Fig. 4A). As shown in volcano map, we can divide the proteins into three types clearly, that are up-regulated, down-regulated and unchanged groups. According to the criterion, 123 DAPs were identified in this study, among them, 70 proteins (56.9 %) were upregulated, and 53 proteins (43.1 %) were downregulated (Fig. 4B). To provide visualization of the overall protein change effect, the DAPs of each groups were analyzed in the form of a heatmap with the hierarchical cluster analysis (Fig. 4C). Moreover, the top 20 proteins of upregulated or downregulated were listed in Table 1.

**Table 1:** The top 20 of up-regulated and down-regulated proteins

Category	Gene symbol	Reference Sequence in NCBI	Protein description	*P-*value
Up	DTD1	XP_017912734.1	D-aminoacyl-tRNA deacylase 1	0.000111266
	SLIRP	XP_005686225.2	SRA stem-loop interacting RNA binding protein	0.000293445
	Alpha-2	XP_005680941.2	Alpha-2-macroglobulin isoform X1	0.000330041
	SOAT1	XP_005690983.1	Sterol O-acyltransferase 1	0.000566201
	RPS27A	XP_005686669.1	Ribosomal protein S27a	0.000577656
	SERINC1	XP_005684572.1	Serine incorporator 1	0.000636264
	SNRPC	XP_005696300.1	Small nuclear ribonucleoprotein polypeptide C	0.000646005
	GPX4	NP_001272641.1	Glutathione peroxidase 4	0.000649723
	SH3BGRL3	XP_017911823.1	SH3 domain binding glutamate rich protein like 3	0.000941752
	FIP1L1	XP_005681673.1	Factor interacting with PAPOLA and CPSF1	0.001017966
	PSIP1	XP_017907427.1	PC4 and SFRS1 interacting protein 1	0.001212773
	LGALS1	XP_017904227.1	Galectin 1	0.00159148
	IRF2BPL	XP_017909416.1	Interferon regulatory factor 2 binding protein like	0.00177422
	CERS5	XP_017903294.1	Ceramide synthase 5	0.001915355
	TRAM2	XP_017894548.1	Translocation associated membrane protein 2	0.00228529
	MBNL1	XP_005675484.1	Muscleblind like splicing regulator 1	0.00235485
	CRTC2	XP_017901639.1	CREB regulated transcription coactivator 2	0.003169094
	TIMP2	XP_017919163.1	TIMP metallopeptidase inhibitor 2	0.003258191
	SRSF10	XP_005676907.2	Serine and arginine rich splicing factor 10	0.003403235
	DAP	XP_017921116.1	Death associated protein	0.004083978
Down	ACTN2	XP_017897988.1	Actinin alpha 2	0.0000082
	ECE1	XP_017910686.1	Endothelin converting enzyme 1	0.00000975
	AIF1L	XP_017911481.1	Allograft inflammatory factor 1 like	0.0000196
	NEXN	XP_017901147.1	Nexilin F-actin binding protein	0.0000232
	MAP3K7CL	XP_005674769.1	MAP3K7 C-terminal like	0.0000386
	COMMD5	XP_005688833.1	COMM domain containing 5	0.0000487
	TNNI2	XP_017898958.1	Troponin I2, fast skeletal type	0.000134909
	DUSP27	XP_017901929.1	Dual specificity phosphatase 27 (putative)	0.000284392
	CKM	XP_005692693.1	Creatine kinase, M-type	0.000331473
	SYNC	XP_017913346.1	Syncoilin, intermediate filament protein	0.000372091
	GPRC5C	XP_017919362.1	G protein-coupled receptor class C group 5 member C	0.000384791
	FBLN1	XP_017904388.1	Fibulin 1	0.000471695
	TNNC1	NP_001272501.1	Troponin C1, slow skeletal and cardiac type	0.000623669
	CSRP3	XP_005699577.1	Cysteine and glycine rich protein 3	0.000752296
	TPT1	XP_017912255.1	Tumor protein, translationally-controlled 1	0.000853458
	MUSTN1	XP_017922589.1	Musculoskeletal, embryonic nuclear protein 1	0.000881408
	TNNI1	XP_017916373.1	Troponin I1, slow skeletal type	0.000995077
	LDB3	XP_017897678.1	LIM domain binding 3	0.001014671
	TNNT2	XP_017915412.1	Troponin T2, cardiac type	0.001035719
	GTF2F1	XP_017906418.1	General transcription factor IIF subunit 1	0.00106987

### GO function and KEGG Pathway enrichment analysis

**Fig. 5:**
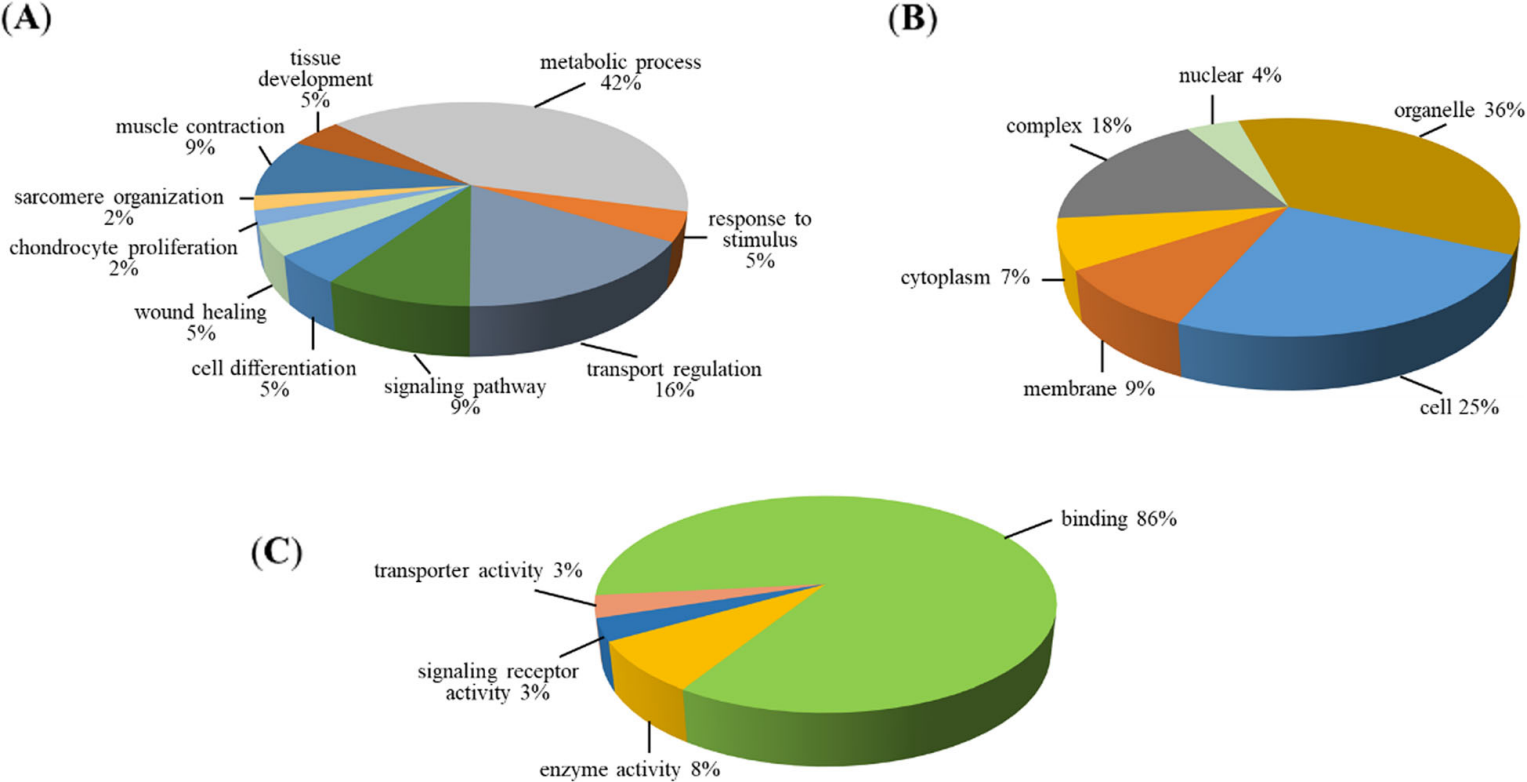
Annotations of the DAPs. **A** GO function enrichment analysis of the DAPs with the top 20 of biological processes. **B** Cellular components of the DAPs. **C** Molecular functions of the DAPs

**Fig. 6:**
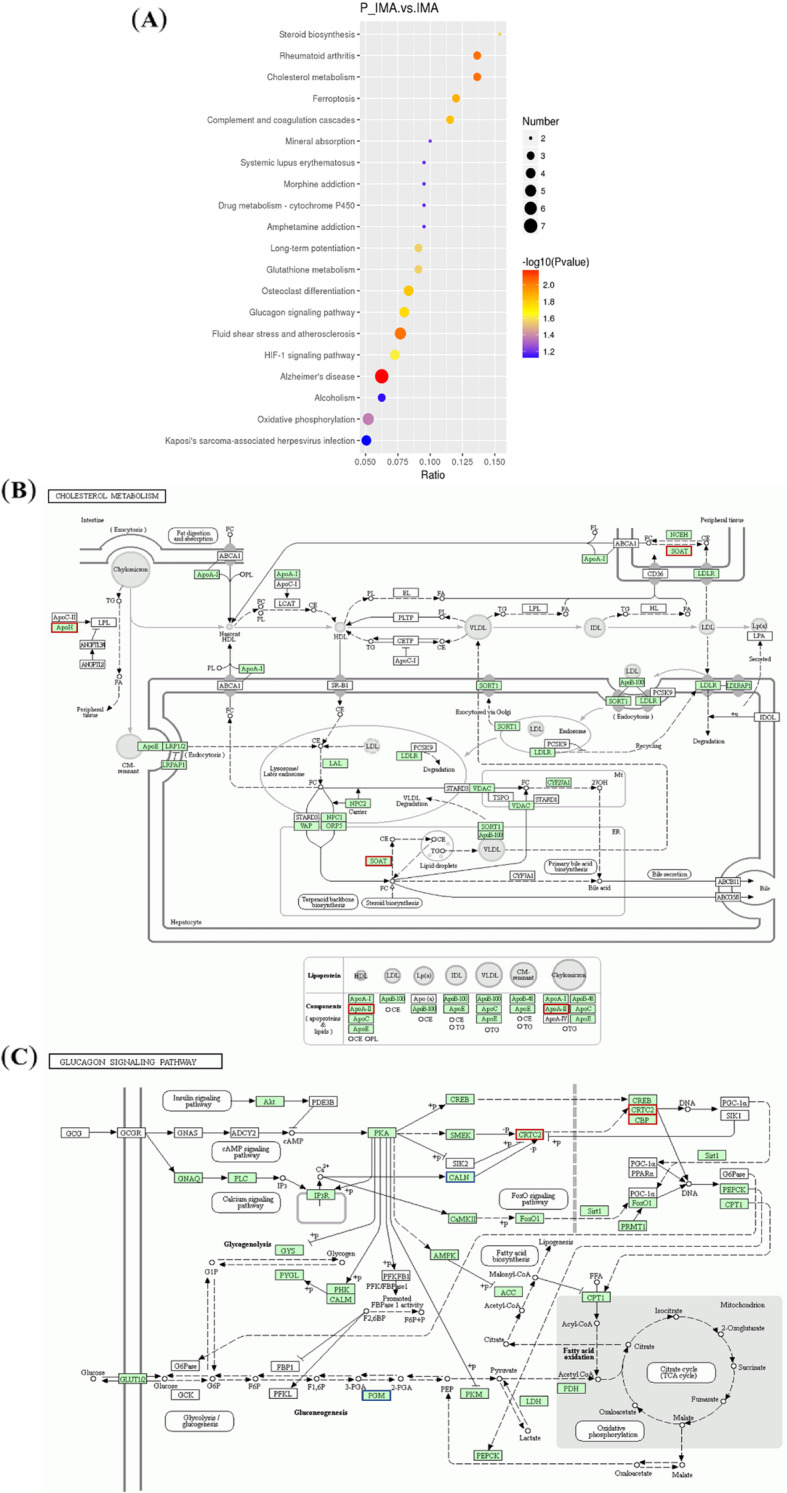
Enrichment analysis of the DAPs. **A** The DAPs were enriched in top 20 most significantly enriched KEGG pathways. **B** Cholesterol metabolism pathway. **C** Glucagon signaling pathway

For further exploring the biological functions of DAPs, we performed GO function annotation analysis on the obtained DAPs, and the top 20 of biological processes, cellular components, and molecular functions were shown in Fig. 5. Using pie chart, we presented the enrichment results, and we found that these DAPs were involved in a variety of biological processes, such as metabolic process (42 %), transport regulation (16 %), signaling pathway (9 %) and muscle contraction (9 %). (Fig. 5A, Supplementary material S2). Cell component analysis showed that these proteins were mainly derived from organelle (36 %), cell (25 %), complex (18 %) and membrane (9 %) (Fig. 5B, Supplementary material S3). Molecular function analysis revealed that 86 % of these DAPs participated in binding and 8 % was involved in enzyme activity (Fig. 5C, Supplementary material S4). Subsequently, KEGG enrichment analysis were performed to investigate the main biochemical metabolic and signal transduction pathways in which DAPs participated. Top 20 pathways of the DAPs mapped were shown in Fig. 6A, (Supplementary material S5). We found that there were 4 proteins (XP_017910927.1, XP_017909416.1, XP_005686153.1 and XP_017908084.1) enriched in kaposi′s sarcoma-associated herpesvirus infection with the lowest *P*-value among identified pathways. Furthermore, three proteins were found enriched in the cholesterol metabolism signaling pathway including beta-2-glycoprotein 1 isoform X1(APOH), apolipoprotein A-II(APOA2) and sterol O-acyltransferase 1(SOAT1) (Fig. 6B). Four proteins enriched in the Glucagon signaling pathway were phosphoglycerate mutase 2(PGAM2), calcineurin subunit B type 1 isoform X1(PPP3R1), interferon regulatory factor 2-binding protein-like (IRF2BPL) and CREB-regulated transcription coactivator 2 isoform X1(CRTC2) (Fig. 6C).

### Network analysis of protein-protein interactions

**Fig. 7:**
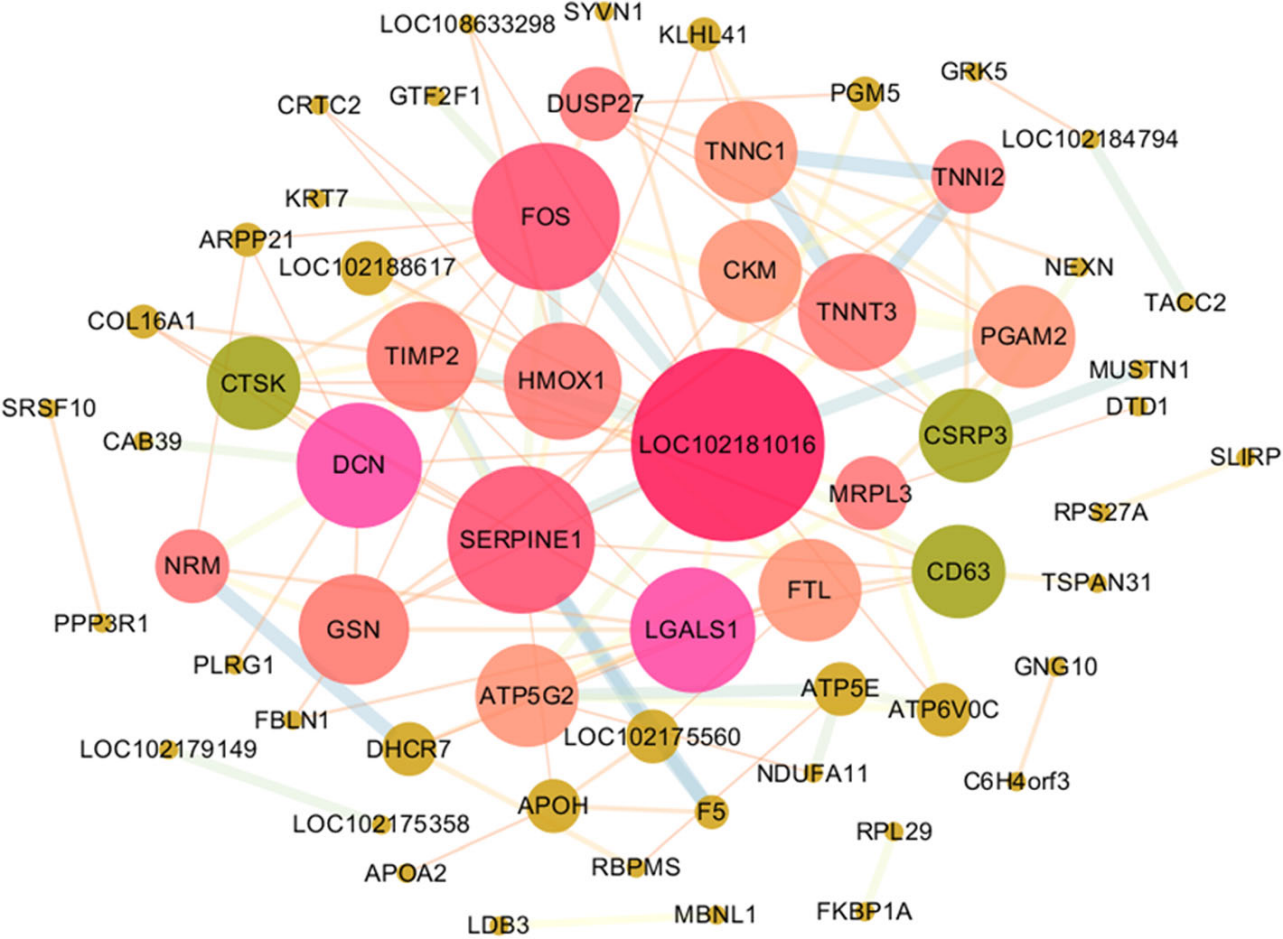
The protein-protein interactions network of DAPs. The bigger network nodes represent the higher the degree of protein interaction and the thickness of the connecting line between nodes represents different connectivity degree

Using StringDB database and Cytoscape software, we constructed a protein-protein interactions (PPI) network for the DAPs. Our results showed that glyceraldehyde-3-phosphate ehydrogenase-like (LOC102181016) exhibited the highest connectivity degree. There are three proteins Fructo-oligosaccharides (FOS / c-Fos), Plasminogen activator inhibitor 1 (PAI-1/SERPINE1) and Phosphoglycerate mutase 2 (PGAM2) exhibiting the highest degree of connectivity with LOC102181016 (Fig. 7, Supplementary material S6).

### PRM Validation of TMT-Based Results

**Fig. 8:**
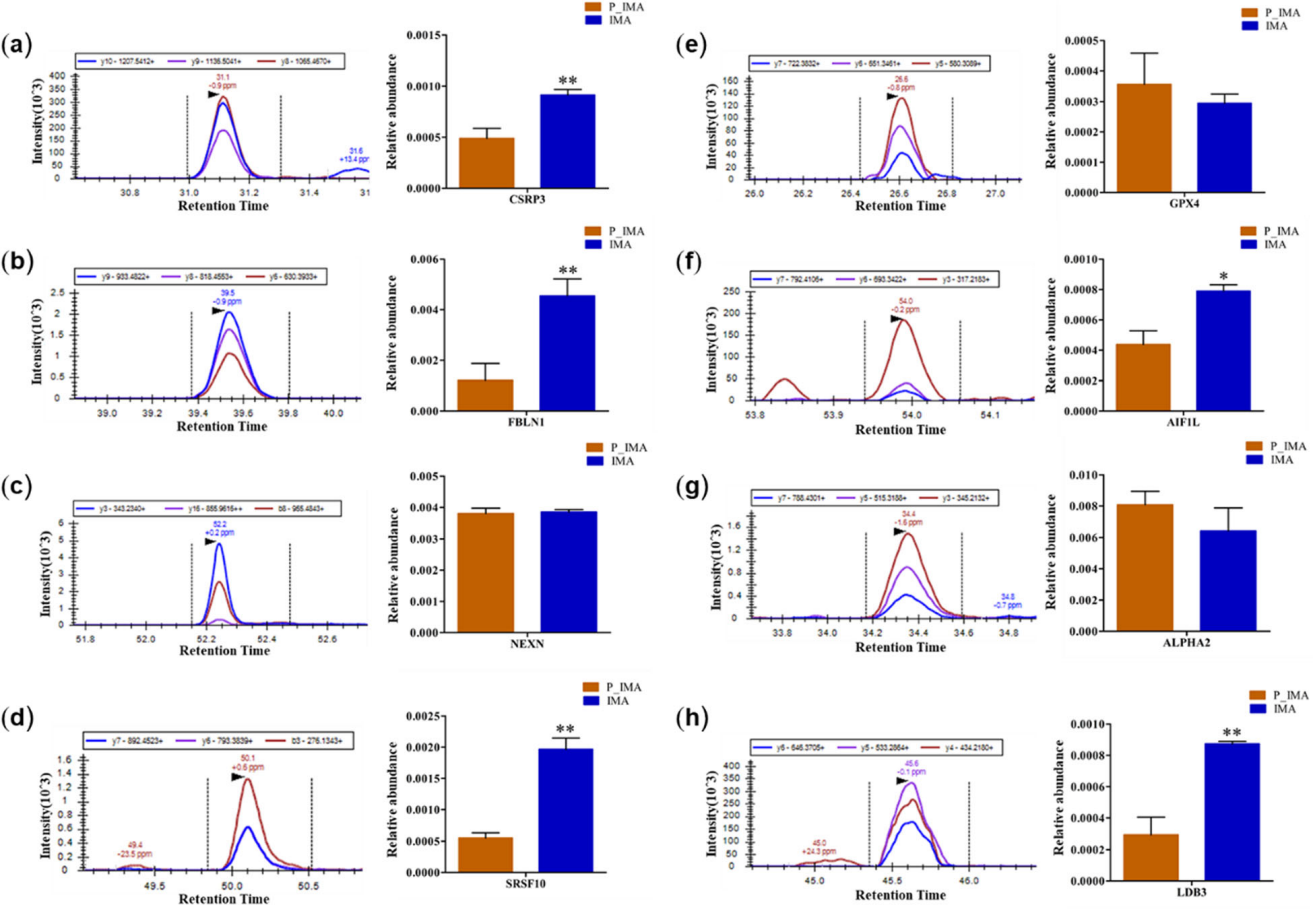
PRM protein expression quantities of the candidate proteins. Three biological replicates per protein. Data are shown as mean ± SD of three samples per protein. * *P* < 0.05; **P < 0.01

To validate the differential proteins between P_IMA and IMA, eight DAPs that may be related to fat formation were selected for verifying by PRM quantitative analysis, which are Cysteine and glycine rich protein 3(CSRP3), Fibulin 1(FBLN1), Nexilin F-actin binding protein(NEXN), Serine and arginine rich splicing factor 10(SRSF10), LIM domain binding 3(LDB3), Alpha-2-macroglobulin isoform X1(ALPHA2), Allograft inflammatory factor 1 like (AIF1L) and Glutathione peroxidase 4(GPX4). According to the quantitative value of the relative expression of the target proteins in the sample, using T-test to calculate the expression difference. After normalized, the results of the relative expression of the proteins were shown in Fig. 8. As shown, there is a significant difference between P_IMA and IMA in the relative enrichment of proteins in CSRP3, FBLN1, SRSF10, LDB3 (*P* < 0.01) and AIF1L(*P* < 0.05). Comparing P_IMA to IMA in this study, CSRP3, FBLN1, SRSF10, LDB3, AIF1L and GPX4 have significant differences in both the TMT and PRM analysis. On the whole, these results are in agreement with the findings in TMT-based quantitative analysis.

## Discussion

Cellular differentiation is a complex process, in which, cells are affected by single-cell communication and the extracellular environment and thus differ from each other in function and morphology. However, one of the final results of cell differentiation is to produce cells with specific functions. For example, skeletal muscle cells produce large amounts of actin and myosin, and red blood cells produce hemoglobin [17–19]. Because of the production of these proteins the cells can perform unique functions. In adipocytes, proteins that promote adipocyte proliferation and lipid deposition are regarded as functional proteins in the process of adipocyte differentiation [18]. Therefore, searching for DAPs during the differentiation process of adipocytes is essential to explore the mechanism of fat deposition.

In this study, a total of 5929 proteins were identified, including 123 DAPs that are 70 upregulated proteins and 53 downregulated proteins. The up-regulated and down-regulated proteins with the smallest *P*-value were DTD1 and ACTN2. The d-tyrosyl-tRNA deacylase 1 (DTD1) plays an important role in metabolic pathways and activation of cellular immune responses [20–22]. The actinin alpha 2 (ACTN2) is one of four encoding isoforms of α-actinin, and genome-wide association and multi-omic analyses reveal ACTN2 as a gene being linked to heart failure [23]. Also, microarray analyses showed that ACTN2 is an important differentially expressed heart-related gene for pig heart steatosis and hypertrophy induced by high-energy diet [24]. Furthermore, the most prominent upregulated protein in our result was serine and arginine rich splicing factor 10 (SRSF10). Previous study declared that *SRSF10* as an essential regulator for adipocyte differentiation could controls the production of *lipin1α* and thus promotes adipocyte differentiation in mice [25]. The most prominent downregulated protein was cysteine and glycine rich protein 3 (CSRP3). CSRP3, highly expressed under insulin-sensitive conditions, was highly inducible protein that plays a key role in regulating glucose homeostasis in skeletal muscle. In addition, knockdown of *CSRP3* suppressed chicken satellite cell differentiation by regulating *Smad3* phosphorylation in the TGF-β signaling pathway [26, 27]. Other research showed that it is related to muscle fiber hypertrophy [28]. These finding suggested that SRSF10 and CSRP3 positively influence the goat IMF differentiation.

GO analysis on the DAPs showed that, most of the DAPs coming from organelles, may perform molecular functions in binding ways and may participate in metabolic processes. Two pathways, cholesterol metabolism and glucagon signaling pathway, were noticed in our study by KEGG pathway enrichment. And both of the two pathways may play essential roles in lipid metabolism and cell differentiation [29–32]. From the KEGG enrichment analysis, we found two pathways may involve in regulating adipocyte differentiation, that were cholesterol metabolism signaling pathway and glucagon signaling pathway. Among them, APOH is involved in lipid metabolism and synthesis [33]. APOA2 is related to obesity, dyslipidemia and lipid metabolism [34, 35]. SOAT1 is involved in atherosclerosis, cholesterol content, glucose and lipid metabolism, study have found that overexpression of *ACAT1/2* encoded by SOAT1 can significantly inhibit the differentiation of 3T3-L1 preadipocytes [36–39]. PGAM2 is a housekeeping enzyme, involved in the process of sugar metabolism, and plays an important role in muscle growth and development [40]. Studies found that PPP3r is a ubiquitously expressed calcium-sensitive serine-threonine phosphatase, and PPP3r KO mice increase energy expenditure. In addition, skeletal muscle specific ablation Ppp3r1 promotes overall number of fat cells per fat pad [41]. IRF2BPL as a transcriptional cofactor, is a new participant in the regulation of cell homeostasis, and also is a new genetic causes for disorders in dystonia [42, 43]. CRTC2, as a critical mediator of mTOR, can induce SREBP-1 processing and enhancement of de novo lipogenesis. mTORC1 regulates the differentiation of beige adipocytes via regulated transcriptional coactivator 2 (CRTC2) [44]. Also, CRTC2 could modulate triglyceride synthesis through regulating of SREBP‐1 maturation [45]. These results suggested the possible signal pathways and proteins involved in goat IMF differentiation.

According to the PPI network, we found LOC102181016 that enriched in glycolysis/gluconeogenesis pathway showed the highest degree of connectivity in DAPs, and glycolysis/gluconeogenesis pathway was a key pathway of IMF deposition [46–48]. The other three strongest interactions with LOC102181016 were FOS, SERPINE1 and PGAM2. FOS belongs to the activator protein 1 (AP-1) superfamily of transcription factors, activation AP-1protein transcription factor Fra-2 in adipocytes in vivo increased differentiation and apoptosis of adipocytes [49, 50]. Moreover, RARγ-C-Fos-PPARγ2 signaling was critical for ATRA-inhibited adipocyte differentiation [51]. SERPINE1 has been reported to be related to adipocyte differentiation, and inhibition of the SERPINE1 in 3T3-L1 adipocytes can increase the expression of PPARγ, promote adipocyte differentiation, and decrease insulin resistance [52, 53]. PGAM2, insulin/IGF1-PI3K-dependent of a glycolytic enzyme, is necessary for nucleolus structure and RNA synthesis [54]. Totally, the exact role of these interacting proteins in goat IMF differentiation still needs to be verified by further experiments.

Using PRM technique, we analyzed the potential proteins for adipogenic differentiation, which exist in top 20 up/down regulated DAPs. According to the results, we found that 5 proteins were differently enriched between goat P_IMA and IMA, that were CSRP3, SRSF10, FBLN1, LDB3 and AIF1L. And the role of CSRP3 and SRSF10 were shown in above discussion. FBLN1 as an extracellular matrix protein, is necessary for osteoblast differentiation in mice. Moreover, it was also secreted in mesenchymal stem cells which derived from human fat [55, 56]. LDB3 was well known for its role in myofibrillar myopathies. Recent study found that the LDB3 promoter responds to lipid uptake in human adipose tissue [57]. AIF1L protein seemed to exist in all adipose tissue. A downregulated expression of AIF1L in intermediate stage of 3T3L1 cell differentiation might limit adipogenesis and/or lipogenesis [58].

## Conclusions

By proteomic comparison between goat P_IMA and IMA, we identified a series protein that might play important role in the goat IMF differentiation, including SRSF10, CSRP3, APOH, PPP3R1, CRTC2, FOS, SERPINE1 and AIF1L. These proteins are of substantial significance for in-depth study of the molecular mechanism of goat IMF differentiation.

## Materials and methods

### Establish the model of goat intramuscular adipocyte differentiation

The samples were collected from the longissimus dorsi of three healthy Jianzhou goats whose age is 7 days old. The experimental animals were injected barbiturate injection into the intraperitoneal cavity at a dose of 100 mg/kg, then bled to death. The carcasses are temporarily stored in freezer and then handed over to a professional solid waste disposal company for unified disposal. The experiment was approved by the Academic Committee of Southwest Minzu University (Chengdu, Sichuan, China), and all the experiment complied with the requirements of the directory of the Ethical Treatment of Experimental Animals of China. Detailed procedure for the collection of intramuscular preadipocytes have previously been published [59, 60].

The goat intramuscular preadipocytes were divided into two groups, each with three biological replicates, and cultured with DMEM/F12 (Hyclone, USA) containing 10 % (v/v) fetal bovine serum (FBS, Hyclone, USA) in 5 % CO2 and 37 °C incubator. The first group preadipocytes, P_IMA, were collected for total protein extracted and RNA extraction when the preadipocytes reached 90 % or more in 6-well plates. The second group, IMA, its medium was replaced with adipogenic inducer of DMEM containing 10 % FBS and 50 µmol•L-1 oleic acid medium, when the preadipocytes reached 90 % in 6-well plates. After 5 days of induction, the cell pellet was collected for total protein extraction and RNA extraction. The medium was refreshed every two days.

### Oil Red O Staining and qRT-PCR

Before staining, the P_IMA and IMA were fixed with 10 % formaldehyde for 30 min then were stained by Oil red O working solution for 20 min. Then the cells were washed by PBS for three times and photographed under microscope. Total RNA was extracted using TRIzol (TaKaRa, Otsu, Japan) and stored at -80℃. RevertAid First Strand cDNA Synthesis Kit (TaKaRa, Otsu, Japan) was used to reverse transcription of mRNA according to manufacturer instructions. Ubiquitously expressed transcript (UXT) was used as endogenous control for mRNA. qRT-PCR primers were designed using Primer Premier 5, and listed in Table 2. Reaction volume for qRT-PCR was 20 µL, including cDNA 1µL, F/R primers 1 µL, double-distilled water 7 µL and 10 µL TB Green™ Premix Ex Taq™ II (TaKaRa, Otsu, Japan).

**Table 2:** Primers utilized in this study. F. Sense primer; R. Antisense primer

Gene	Reference in GenBank	Primer sequence (5′ − 3′)	Tm/°C
*PPARg*	NM_001285658.1	F: AAGCGTCAGGGTTCCACTATG R: GAACCTGATGGCGTTATGAGAC	60℃
*LPL*	NM_001285607.1	F: TCCTGGAGTGACGGAATCTGT R: GACAGCCAGTCCACCACGAT	60℃
*UXT*	XP_005700899.1	F: GCAAGTGGATTTGGGCTGTAAC R: TGGAGTCCTTGGTGAGGTTGT	60℃

### TMT sample preparation

The cells of PIMA and IMA we collected and minced individually in liquid nitrogen and lysed with lysis buffer containing 0.2 % SDS, 6 M Urea and 100 mM NH4HCO3 (pH 8), followed by ultrasonication on ice for 5 min. Subsequently, the supernatant was transferred into a clean tube, and mixed with 2 mM DTT for 1 h at 56 °C and subsequently alkylated with enough Iodoacetamide for 1 h at room temperature in the dark. Then 4 times volume of pre-cooled acetone was added to the samples and incubated at -20 °C for 2 h. The sample was centrifuged at 12 000 rpm for 15 min at 4 °C and the precipitation was collected. After washing pellet twice with cold acetone, it was dissolved by dissolution buffer solution containing 6 M urea and 0.1 M triethylammonium bicarbonate (TEAB, pH 8.5). The protein concentration was determined by the Bradford protein assay (Bio-Rad, USA). The 12 % SDS-PAGE electrophoresis was performed for 20 µg of proteins extracted from each sample with loading buffer, and the gel was stained by Coomassie Blue R-250 for showing the protein bands.

The 120 µg protein of each sample, whose final volume of 100 µL with dissolution buffer solution, was digested with 3µL of Trypsin Gold (1 µg/µL, Promega, USA) and 500 µL of TEAB buffer (50 mM) at 37 °C during one night. Adding the same volume of 1 % formic acid, the digestion mixture was centrifuged at 12 000 rpm for 5 min. The supernatant was desalted with C18 cartridge and washed three times by 1 mL of cleaning solution containing 0.1 % formic acid and 4 % acetonitrile, followed by washing twice with 0.4 mL of eluent containing 0.1 % formic acid and 75 % acetonitrile for removing the high urea and finally was dried by vacuum centrifugation.

The desalted peptides were labeled with TMT 6-plex reagents (Thermo Fisher Scientific, USA), following the manufacturer’s instructions. In brief, 1 unit of labeling reagent was used for 0.1 mg of peptide. The Peptides, dissolved in 100 µL of 0.1 M TEAB, were incubated with the labeling reagent, which was dissolved in 41 µL of acetonitrile, for 2 h at room temperature. Then, three pooled fractions of goat intramuscular preadipocytes group have been labeled with 126, 127, and 128 tags, while those of goat intramuscular adipocytes induced at Day 5 group have been labeled with 129, 130, and 131 tags, individually. The reaction was stopped with 8 % ammonium hydroxide. The differently labeled peptides were mixed equally and desalted by peptide desalting spin columns (Thermo Fisher, USA).

Mobile phases A containing 2 % acetonitrile and the ammonium hydroxide (pH 10.0) and B containing 98 % acetonitrile and the ammonium hydroxide (pH 10.0) were used for developing a gradient elution. The TMT-labeled peptides were dissolved in 1 mL of phases A. The 1 mL supernatant was fractionated using a C18 column (4.6 × 250 mm, 5 μm) on a L3000 HPLC (Rigol, China) and the column oven was set as 50 °C. The solvent gradient was listed as follows: 97 % A and 3 % B for 0 min; 95–97 % A and 3–5 % B for 10 min; 80–95 % A and 5–20 % B for 20 min; 60–80 % A and 20–40 % B for 18 min; 50–60 % A and 40–50 % B for 2 min; 30–50 % A and 50–70 % B for 3 min; 0–30 % A and 70–100 % B for 1 min; 0 % A and 0-100 % B for 4 min, and 0 % A and B for 12 min. The eluates were collected once in 1 min and merged into 10 fractions finally, which were monitored at UV 214 nm. All fractions were dried under vacuum and re-dissolved in 0.1 % formic acid.

### LC-MS/MS analysis

The shotgun proteomics analysis was performed by the EASY-nLCTM 1200 UHPLC system (Thermo Fisher Scientific, USA) coupled with the Orbitrap Q Exactive HF-X mass spectrometer (Thermo Fisher Scientific, USA). The sample with 2 µg of the total peptides was injected onto the homemade pre-column (2 cm×100 μm, 5 μm). Then, the peptides were separated using the homemade analytical column (15 cm×150 μm, 1.9 μm) in 90 min at a flow rate of 600 nL/min, with the eluent B (0.1 % formic acid in 80 % acetonitrile) in eluent A (0.1 % formic acid in H2O) for the TMT labeling 6-plex. The solvent gradient was set as follows: 95 % A and 5 % B for 0 min; 90–95 % A and 5–10 % B for 2 min; 60–90 % A and 10–40 % B for 80 min; 45–60 % A and 40–55 % B for 2 min; 10–45 % A and 55–90 % B for 1 min, and 0–10 % A and 90–100 % B for 5 min. The Orbitrap Q Exactive HF-X mass spectrometer was operated in the data-dependent acquisition mode with the spray voltage of 2.3 kV and capillary temperature of 320 °C, when the Nanospray Flex™ as the ion source. The full-scan range from 350 to 1500 m/z was acquired with a mass resolution of 6 × 104 at 200 m/z. The automatic gain control (AGC) target was set as 3 × 106, while the maximum ion injection time was 20 ms. The 40 most abundant precursor ions selected from the full-scan were fragmented using the higher energy collisional dissociation (HCD) for the MS/MS scans. The MS/MS scans were set as follows: a mass resolution of 1.5 × 104 at 200 m/z, an AGC target value of 1 × 105, a maximum ion injection time of 45 ms, and a normalized collision energy of 32 %.

The obtained spectrums were searched against NCBI nr database (X101SC19051573-Z01-Capra-hircus) by the search engines: Proteome Discoverer 2.2 (PD 2.2, Fisher Scientific, USA). A mass tolerance as10 ppm for precursor ion scan and a mass tolerance as 0.02 Da for the product ion was set as the searched parameter. For reducing the false positive rate and increasing the quality of analysis results, the confident peptide spectrums were matched that confidence more than 99 % while false discovery rate (FDR) less than 1 %. Also, the distribution of the peptide length, precursor ion tolerance, unique peptides number, protein coverage, and protein mass were regarded as the key indicators for the verification of total protein quality. The differentially expressed proteins in this study were defined based on the following criteria: the TMT ratio being > 1.5 or < 0.67 with a *P*-value < 0.05. While T-Test was used to analyze the proteins quantitation results statistically [61, 62].

### Bioinformatics analysis of DAPs

The GO analysis was performed by interproscan-5 program against the non-redundant protein databases [63]. According the biological processes, cellular components, and molecular functions, DAPs are classified. Moreover, the DAPs pathways were annotated using the KEGG database, a collection of databases dealing with genomes, diseases, biological pathways, drugs, and chemical materials (http://www.genome.jp/kegg/) [64, 65]. Based on the goat (*Capra hircus*) species, the potential interacting partners of DAPs were predicted by the StringDB server (http://string-db.org/) [66]. Using Cytoscape software to visualize the prediction results obtained from the StringDB database.

### Parallel reaction monitoring validation

To verify the results of TMT analysis coupled with LC-MS/MS, the proteins were extracted and enzymatically hydrolyzed according to the above method. Same amount of trypsin treated-peptide was taken of each sample, and labeled peptide DSPSAPVNVTVR (red bold V for heavy isotope labeling) as an internal standard of each sample and the peptides were dissolved in 0.1 % formic acid (solvent A) and solvent B (0.1 % formic acid in 80 % acetonitrile). PRM mass spectrometric analysis was performed using EASY-nLCTM 1200 UHPLC system (Thermo Fisher Scientific, USA). Liquid chromatography elution gradient was showed in Table 3. The full scan mass spectrum resolution was set to 60,000 (200 m/z), the maximum C-trap and IT were 3×10^6^ and 20 ms respectively. The PRM resolution was set to 30,000(200 m/z), the maximum C-trap and IT were 5×10^4^ and 80 ms respectively. Normalized collision energy is 27. The data was analyzed by Skyline software, and the peak area was corrected using the internal standard peptide.

**Table 3:** Liquid chromatography elution gradient table

Time (min)	Flow rate (nL/min)	Mobile phase A (%)	Mobile phase B (%)
0	600	94	6
2	600	90	10
49	600	70	30
52	600	65	35
54	600	50	50
55	600	0	100
60	600	0	100

## Supplementary Information

**Additional file 1: Supplementary material S1. **Totally proteins found in two samples.

**Additional file 2: Supplementary material S2.** GO functional enrichment analysis of biological processes involved in DAPs.

**Additional file 3: Supplementary material S3.** GO functional enrichment analysis of cell component involved in DAPs.

**Additional file 4: Supplementary material S4.** GO functional enrichment analysis of molecular function involved in DAPs.

**Additional file 5: Supplementary material S5.** KEGG enrichment analysis the main biochemical metabolic and signal transduction pathways of the DAPs.

**Additional file 6: Supplementary material S6.** The data of protein-protein interactions.

## Data Availability

The datasets generated during the current study are deposited in Capra hircus -- NCBI (https://www.ncbi.nlm.nih.gov/protein).

## Abbreviations

P_IMA: Intramuscular preadipocytes
IMA: Intramuscular adipocytes
IMF: Intramuscular fat
DAPs: Differentially abundant proteins
TMT: The tandem mass tag
LC-MS/MS: Liquid chromatography mass spectrometry analysis
PRM: Parallel Reaction Monitoring
GO: Gene Ontology
KEGG: Kyoto Encyclopedia of Genes and Genomes
qRT-PCR: Quantitative real time PCR
*PPARg*: Preoxisome proliferator-activated receptor gamma
*LPL*: Lipoprotein lipase
